# Supplementary material for: Multi‐dataset identification of innovative feature genes and molecular mechanisms in keratoconus
Source: J Cell Mol Med. 2024 Sep 19;28(18):e70079. doi: 10.1111/jcmm.70079 (PMC11412914; doi:10.1111/jcmm.70079)
Supplement: Supplementary file 2 — Material S2. [file JCMM-28-e70079-s002.zip › 7.GSEA analysis/1.ARL11/KEGG_ALANINE_ASPARTATE_AND_GLUTAMATE_METABOLISM.html]

Details for gene set KEGG\_ALANINE\_ASPARTATE\_AND\_GLUTAMATE\_METABOLISM[GSEA]

|  || Dataset | ARL11.group.cls#h\_versus\_l |
| Phenotype | group.cls#h\_versus\_l |
| Upregulated in class | h |
| GeneSet | KEGG\_ALANINE\_ASPARTATE\_AND\_GLUTAMATE\_METABOLISM |
| Enrichment Score (ES) | 0.2771276 |
| Normalized Enrichment Score (NES) | 0.92803496 |
| Nominal p-value | 0.56647396 |
| FDR q-value | 1.0 |
| FWER p-Value | 1.0 |
Table: GSEA Results Summary

  

Fig 1: Enrichment plot: KEGG\_ALANINE\_ASPARTATE\_AND\_GLUTAMATE\_METABOLISM      
 Profile of the Running ES Score & Positions of GeneSet Members on the Rank Ordered List

  

| SYMBOL | TITLE | RANK IN GENE LIST | RANK METRIC SCORE | RUNNING ES | CORE ENRICHMENT || 1 | GPT | na | 467 | 0.485 | 0.1510 | Yes |
| 2 | GAD1 | na | 1955 | 0.300 | 0.2099 | Yes |
| 3 | AGXT2 | na | 4162 | 0.195 | 0.2125 | Yes |
| 4 | IL4I1 | na | 4200 | 0.194 | 0.2771 | Yes |
| 5 | DDO | na | 7947 | 0.093 | 0.2009 | No |
| 6 | PPAT | na | 9290 | 0.070 | 0.1862 | No |
| 7 | GOT2 | na | 9613 | 0.066 | 0.1991 | No |
| 8 | GLS2 | na | 11078 | 0.047 | 0.1728 | No |
| 9 | GLUD1 | na | 12533 | 0.031 | 0.1415 | No |
| 10 | ALDH4A1 | na | 12945 | 0.028 | 0.1390 | No |
| 11 | ASL | na | 13160 | 0.026 | 0.1415 | No |
| 12 | GLUL | na | 13199 | 0.025 | 0.1489 | No |
| 13 | GAD2 | na | 15039 | 0.011 | 0.0996 | No |
| 14 | NIT2 | na | 15508 | 0.007 | 0.0887 | No |
| 15 | CAD | na | 16412 | 0.001 | 0.0629 | No |
| 16 | ASNS | na | 16835 | -0.002 | 0.0515 | No |
| 17 | ASS1 | na | 17005 | -0.003 | 0.0477 | No |
| 18 | ABAT | na | 17429 | -0.006 | 0.0377 | No |
| 19 | GOT1 | na | 19508 | -0.021 | -0.0153 | No |
| 20 | ADSL | na | 19975 | -0.024 | -0.0207 | No |
| 21 | GFPT2 | na | 21277 | -0.033 | -0.0469 | No |
| 22 | GLS | na | 21462 | -0.035 | -0.0403 | No |
| 23 | GPT2 | na | 23216 | -0.052 | -0.0730 | No |
| 24 | AGXT | na | 24023 | -0.063 | -0.0750 | No |
| 25 | ALDH5A1 | na | 25959 | -0.090 | -0.1004 | No |
| 26 | CPS1 | na | 26381 | -0.096 | -0.0798 | No |
| 27 | GLUD2 | na | 26662 | -0.102 | -0.0533 | No |
| 28 | GFPT1 | na | 27611 | -0.121 | -0.0395 | No |
| 29 | ACY3 | na | 32177 | -0.268 | -0.0800 | No |
| 30 | ASPA | na | 34196 | -0.452 | 0.0153 | No |
Table: GSEA details [plain text format]

  

Fig 2: KEGG\_ALANINE\_ASPARTATE\_AND\_GLUTAMATE\_METABOLISM      
 Blue-Pink O' Gram in the Space of the Analyzed GeneSet

  

Fig 3: KEGG\_ALANINE\_ASPARTATE\_AND\_GLUTAMATE\_METABOLISM: Random ES distribution      
 Gene set null distribution of ES for **KEGG\_ALANINE\_ASPARTATE\_AND\_GLUTAMATE\_METABOLISM**
